# FGF4 and Retinoic Acid Direct Differentiation of hESCs into PDX1-Expressing Foregut Endoderm in a Time- and Concentration-Dependent Manner

**DOI:** 10.1371/journal.pone.0004794

**Published:** 2009-03-11

**Authors:** Martina Johannesson, Anders Ståhlberg, Jacqueline Ameri, Fredrik Wolfhagen Sand, Karin Norrman, Henrik Semb

**Affiliations:** 1 Lund Center for Stem Cell Biology and Cell Therapy, Lund University, Lund, Sweden; 2 Department of Clinical Neuroscience and Rehabilitation, Institute of Neurosciences and Physiology, Sahlgrenska Academy at Göteborg University, Göteborg, Sweden; Istituto Dermopatico dell'Immacolata, Italy

## Abstract

**Background:**

Retinoic acid (RA) and fibroblast growth factor 4 (FGF4) signaling control endoderm patterning and pancreas induction/expansion. Based on these findings, RA and FGFs, excluding FGF4, have frequently been used in differentiation protocols to direct differentiation of hESCs into endodermal and pancreatic cell types. In vivo, these signaling pathways act in a temporal and concentration-dependent manner. However, in vitro, the underlying basis for the time of addition of growth and differentiation factors (GDFs), including RA and FGFs, as well as the concentration is lacking. Thus, in order to develop robust and reliable differentiation protocols of ESCs into mature pancreatic cell types, including insulin-producing β cells, it will be important to mechanistically understand each specification step. This includes differentiation of mesendoderm/definitive endoderm into foregut endoderm- the origin of pancreatic endoderm.

**Methodology/Principal Findings:**

Here, we provide data on the individual and combinatorial role of RA and FGF4 in directing differentiation of ActivinA (AA)-induced hESCs into *PDX1*-expressing cells. FGF4's ability to affect endoderm patterning and specification in vitro has so far not been tested. By testing out the optimal concentration and timing of addition of FGF4 and RA, we present a robust differentiation protocol that on average generates 32% PDX1^+^ cells. Furthermore, we show that RA is required for converting AA-induced hESCs into PDX1^+^ cells, and that part of the underlying mechanism involves FGF receptor signaling. Finally, further characterization of the PDX1^+^ cells suggests that they represent foregut endoderm not yet committed to pancreatic, posterior stomach, or duodenal endoderm.

**Conclusion/Significance:**

In conclusion, we show that RA and FGF4 jointly direct differentiation of PDX1^+^ foregut endoderm in a robust and efficient manner. RA signaling mediated by the early induction of RARβ through AA/Wnt3a is required for PDX1 expression. Part of RA's activity is mediated by FGF signaling.

## Introduction

To achieve the goal of creating a practical, replenishable source of β cells for transplant therapy of patients with Type 1 diabetes, it will be critical to understand the embryonic processes that generate β cells, and to translate this knowledge into human cellular systems.

Pancreatic β cells develop by progressive instructive differentiation of pancreatic progenitors, which are derived as a result of the regionalized differentiation of the definitive endoderm (DE). Before any morphological signs of organogenesis are apparent in the primitive gut tube, the endoderm becomes patterned through the actions of a complex cross talk between mesoderm and endoderm involving gradients of fibroblast growth factors (FGFs), bone morphogenic proteins (BMPs), retinoic acid (RA), and sonic hedgehog (SHH) [1]–[3]. Initially, the pancreas forms as a ventral and a dorsal bud. The ventral bud is surrounded by cardiac mesoderm and the dorsal bud is in contact with the notochord and subsequently the dorsal aorta. Those are all mesodermally derived tissues that influence formation of the pancreas [4], [5].

Human embryonic stem cells (hESC) are derived from the inner cell mass (ICM) of the blastocyst and have the potential to in vitro follow the same developmental pathways as the ICM, including differentiation into pancreatic cells [6]. Since the pancreas, including the endocrine component, is derived from DE, there have been focused efforts on in vitro induction of early endodermal cell types. This approach has been taken in a number of recent studies where knowledge of the signaling events that orchestrate primitive streak (PS) formation, gastrulation, and formation of DE during early mouse development has been employed [7]–[9]. Although ESC-derived endoderm can be further differentiated into more mature cell types, such as liver and pancreatic cells [7], [8], [10]–[13], robust experimental strategies to pattern DE into posterior foregut endoderm and multipotent pancreatic endoderm are lacking.

Retinaldehyde dehydrogenase (Raldh2), the enzyme responsible for the biosynthesis of RA, is expressed in mesoderm during gastrulation where it has been shown to pre-pattern the endoderm and regulate early stages of pancreas development [14], [15]. Raldh2 is also expressed in the mouse dorsal pancreatic mesenchyme at the early stage of pancreas specification until E12.5 [16], [17]. In addition, RA acts as a posteriorizing agent in the gut endoderm. In embryos with increased RA signaling, pancreas and liver fates were expanded rostrally at the expense of anterior endoderm fates such as thyroid and pharynx. Pancreas and liver specification requires RA signaling, but more posterior endodermal organs do not, implying a subdivision of the endoderm into RA-responsive and non-responsive domains by late gastrulation. Several retinoic acid receptors (RARs) exist of which RARβ is the primary target for RA [18]. RARs are ligand-activated transcription factors that bind to retinoic acid response elements (RAREs) within the promoter of their target genes. In embroid bodies (EBs) from mESC, RA induces pancreatic duodenal homeobox 1 (PDX1/IPF1)^+^ pancreatic endoderm [12]. Pdx1 is a main regulator of pancreas specification and β cell function [19]–[21]. RA is often included in multi-factorial differentiation protocols towards pancreatic cell types, albeit without defining its exact role. Moreover, to our knowledge, the expression of RARs has not been studied during differentiation of embryonic stem cells towards DE and pancreatic cell types.

FGF4, which is expressed in the vicinity of the posterior endoderm in the gastrula and early somite stage embryos, exhibit a broad anterior-posterior patterning activity in the gut endoderm. Specifically, FGF4 promotes posterior and inhibits anterior endoderm cell fate [22]. FGF4 signals mainly via FGFR1c and FGFR2c and to a smaller extent via FGFR3c and FGFR4 [23]. Importantly, moderate levels of FGF4 are needed to maintain *Pdx1* expression, whereas high levels of FGF4 repress *Pdx1* expression. Thus, this data suggests that endoderm is patterned by FGF4 both in a concentration and in a temporal dependent manner and that the pancreas arise from cells that receive intermediate levels of FGF4 [24]. Importantly, whether FGF4 affect ESC-derived DE in a similar manner remains unknown.

Other FGFs, such as FGF1 and FGF2 that are produced by the cardiac mesoderm, are also involved in gut endoderm patterning, albeit in a more restricted manner. These FGFs pattern the foregut endoderm in a concentration-dependent manner, i.e. at lower concentrations liver fate is promoted, whereas at higher concentrations lung fate is promoted [25].

Notably, RA and FGF signaling, which both exhibit endodermal patterning activities and support pancreas specification, seem to cross talk during these events [26]. For example, RAR is required for the correct expression of fgf8, fgfr1 and fgfr4, and addition of endogenous RA induces expression of fgf8, fgfr1 and fgfr4 in animal cap experiments. Moreover, *XCAD3* (the equivalent of mammalian *Cdx4*) is a key downstream gene in the FGF-mediated posteriorization pathway and retinoids are known to influence the expression of caudal genes in other systems [27].

Here, we test the ability of RA and FGF4 alone and in combination to direct differentiation of hESC-derived DE into PDX1^+^ posterior foregut endoderm. By optimizing the timing and concentration of RA and FGF4, approximately 30% of all cells turn into PDX1^+^ foregut endoderm. Furthermore, RA is required for differentiation into PDX1^+^ cells and part of its activity is mediated by FGF signaling, suggesting cross talk between RA and FGF signaling during RA-induced foregut specification from hESC.

## Methods

### Human embryonic stem cell culture

#### Routine culture

The hESC lines Hues-1, Hues-3 and Hues-15 were obtained from D.A. Melton, Howard Hughes Medical Institute (Harvard institute, Cambridge, MA) and cultured according to protocols at http://mcb.harvard.edu/melton/hues/ as previously described [28]. Whereas the RA/FGF4 protocol was tested on Hues-3 (subclone 52) and Hues-15, the D'Amour protocol was tested on Hues-1 and Hues-3. The Cells were maintained in KO-DMEM (Gibco) supplemented with 10% KO serum replacement (Gibco), 1% Non-essential amino acids (Gibco), 1% Glutamax (Gibco), 0.1% beta-mercaptoethanol (Gibco), 1% penicillin-streptomycin (PEST) (Invitrogen), 10% plasmanate (Talecris), and 10 ng/mL bFgf (Invitrogen). The medium was changed daily to keep the cells in an undifferentiated state. Cells were passaged with 0.05% trypsin-EDTA (Gibco) every third or fourth day onto freshly seeded (25,000/cm^2^) mitotically inactivated mouse embryonic feeder-cells (MEFs) (Sahlgrenska Akademin Experimental Biomedicine University of Gothenburg) at a density of 12,000 cells/cm^2^ for Hues-3 (subclone 52) and 30,000 cells/cm^2^ for Hues-15. The cell lines were karyotyped by standard G-banding by the Institute of Clinical Genetics at the University of Linköping, Sweden. 12–23 metaphases were evaluated. Hues-1 and Hues-15 were found to be karyotypically normal, whereas Hues-3 (subclone 52) has a gain of material from chromosome 17 (82%).

#### Differentiation experiments

**Figure 1:**
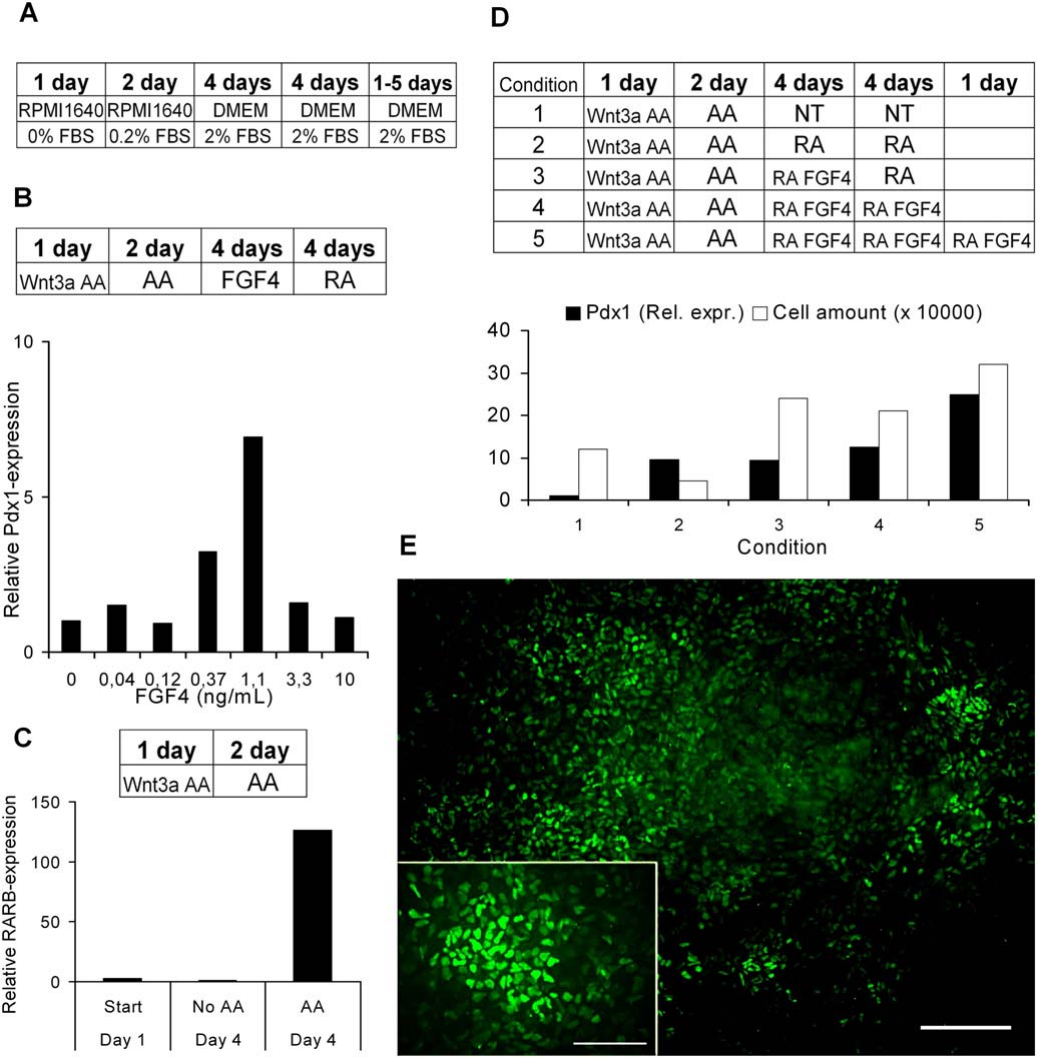
Overview of the different experiments leading up to conditions for obtaining *PDX1*-positive cells. (A) Medium compositions in the differentiation procotol. (B) Optimal concentration of fibroblast growth factor 4 (FGF4) on days 4–7 in the presence of retinoic acid (RA) on days 8–11. (C) mRNA expression of the retinoic acid receptor beta, *RARβ*, after activin (AA) induction. (D) The impact of FGF4 and RA on relative *PDX1* gene expression (Rel. Expr.) and cell amount. Abbreviations and concentrations used: AA = Activin A (100 ng/mL), Fgf4 (1.1 ng/mL) where not stated otherwise, RA = Retinoic acid (2 µM), NT = no treatment after activin induction. (E) Immunofluorescence staining of Pdx1 using Pdx1-anti-goat (1∶1500) on day 13. Scale bars: E, 100 µm ; inset, 200 µm.

For differentiation experiments, Hues-3 (subclone 52) cells were seeded at a density of 20,000 cells/cm^2^ at passages 68–76, and cultured for three to four days until a confluent flat layer of undifferentiated cells was formed. Hues-15 cells were seeded at a density of 17,000 cells/cm^2^ at passage 23. At the start of each differentiation procedure at high confluence of the cells, phosphate buffered saline (PBS) (Gibco) was used to wash the cell layer once. The medium-composition during the differentiation experiments is described in Fig. 1A. Activin A (100 ng/mL) (R&D systems) and Wingless-type MMTV integration site family, member 3A (Wnt3a) (25 ng/mL) (R&D systems) was used to induce definitive endoderm (DE) in Rosewell Park Memorial Intitute (RPMI) 1640 (Gibco) supplemented with no fetal bovine serum (FBS) (Sigma) the first day and 0.2% FBS the second and third day. As a control for DE-induction, RPMI 1640 was used without addition of substances other than FBS. At day four, samples were taken for real-time polymerase chain reaction (PCR) analysis. On days four to seven, RPMI 1640 was supplemented with 2% FBS and from day eight, Dulbecco's modified Eagle medium (DMEM) (Gibco) was used supplemented with 2% FBS. From day four onward, Fibroblast growth factor 4 (FGF4) (R&D systems), and Retinoic acid (RA) (Sigma) were added in different combinations and concentrations as described. On various different time-points, cyclopamine (Sigma) was used in a concentration of 0.25 µM in order to inhibit shh. Penicillin/Streptomycin (PEST) (1%) was added to the differentiation medias. Non-treated (NT) cells did not get addition of substances other than DE-induction. For the D'Amour protocol, cells were treated as previously described [7]. When the D'Amour protocol was tested on cell line Hues-1, cells died at stage three representing the posterior foregut stage. However, with cell line Hues-3, a small number of PDX1^+^ cells was obtained at stage three. Still, cells did not survive further treatment onto stage four (pancreatic and endocrine precursors) or five (hormone expressing endocrine cells) (data not shown). Brightfield images of cells were taken on an inverted microscope (Eclipse TE2000-U) (Nicon).

### mRNA extraction and reverse transcription

Cells were harvested after trypsinization (0.05% Trypsin-EDTA) and purified according to the protocol of GeneElute Mammalian Total RNA Miniprep kit (Sigma). The mRNA concentrations were determined by a NanoDrop ND-1000 spectrophotometer (Saveen Werner). The reverse transcription was performed with SuperScript III (Invitrogen). Initially, mRNA (50–500 ng), 2 µM random hexamers (Invitrogen), 2 µM Oligo (dT) (Invitrogen), and 10 mM deoxynucleotidetriphosphates (dNTP) (Fermentas) were incubated at 65°C for five minutes followed by cooling down to 8°C. In the second step, 1×First Strand (FS) buffer (Invitrogen), 5 mM DTT (Invitrogen), 10 U Superscript™ III Reverse transcriptase (Invitrogen), and 2 U RNaseOUT™ (Invitrogen) was added to a final reaction volume of 10 µL. The temperature profile of the reverse transcription reaction was 25°C for five minutes, 50°C for 45 minutes, 55°C for 10 minutes, and 70°C for 15 minutes. All samples were diluted to 200 µL with water and stored in −20°C for later analysis by real-time PCR.

#### Reverse transcription-polymerase chain reaction (RT-PCR) analysis

Primers for RT-PCR were designed using Primer 3 (http://www-genome.wi.mit.edu/cgi-bin/primer/primer3_www.cgi) and are shown in Table 1. Assays for some pancreatic progenitor markers, such as Ptf1a and Nkx6.1, were designed and confirmed on human pancreatic tissue as a positive control (data not shown). The pancreatic tissue was kindly provided by O. Korsgren at the University of Uppsala, Sweden. Human islets were used for cDNA-synthesis in order to analyse the relative mRNA expression of *PDX1* in islets compared to cells differentiated according to the RA/FGF4-protocol on day 16. Altogether, twelve RA/FGF4-treated samples from day 16 in experiments 1–3 were compared to human islet cDNA. Real-time PCR was carried out using the 7900HT Fast Real-time PCR system (Applied biosystems). SYBR green was used as a double-stranded DNA-specific fluorescent dye as detection chemistry in the real-time PCR. Reactions were performed with the following constituents: forward and reversed primers 400 nM, 1×Platinum® Quantitative PCR superMix-UDG with ROX (Invitrogen), 0.125×SYBR (Invitrogen), and 3 µL template cDNA in 20 µL reactions. The PCR was performed using the following settings: Preincubation at 50°C for 2 min, and 95°C for 2 min followed by 45 cycles with denaturation at 95°C for 15 sec, annealing at 60°C for 25 sec, and extension at 73°C for 30 sec. Cycle of threshold (C_t_)-values were determined using manual C_t_ and automatic baseline. The correct PCR-product was confirmed by agarose gel electrophoresis (2% w/v) and melting curve analysis. Data analysis and relative quantification was performed as described [29], [30], using an overall PCR efficiency of 90%. Data was normalized against *ACTB*. *ACTB* was verified as a suitable reference gene by GeNorm [31]. The lowest value in each data set was arbitrarily set to one and the rest of the data points were related to this value. In cases where no gene expression was measured, as for some genes of non-treated control cells, Ct values were set to 45, i.e. the maxium amount of cycles run. Each experiment was performed three times to confirm data. In each experiment, two to four biological replicates were measured. In addition, duplicate technical replicates were used throughout the measurements. Mean values±SEM were calculated.

**Table 1:** Primer sequences used in RT-PCR.

Gene	Forward primer sequence	Reversed primer sequence
ACTB	5′-CTGGAACGGTGAAGGTGACA-3′	5′- AAGGGACTTCCTGTAACAATGCA-3′
PDX1	5′-CCCATGGATGAAGTCTACC-3′	5′- GTCCTCCTCCTTTTTCCAC-3′
SOX9	5′-GAGGAAGTCGGTGAAGAACG-3′	5′-CCAACATCGAGACCTTCGAT-3
HNF6	5′-CGGAGGATGTGGAAGTGG-3′	5′-TTTGGATGGACGCTTATTTTC-3′
FOXA2	5′-AGGAGGAAAACGGGAAAGAA-3′	5′-CAACAACAGCAATGGAGGAG-3′
CDX2	5′-ACCTGTGCGAGTGGATGC-3′	5′-TCCTTTGCTCTGCGGTTCT-3′
CXCR4*	5′-CACCGCATCTGGAGAACCA-3′	5′-GCCCATTTCCTCGGTCTAGTT-3′
SOX17	5′-AAGGGCGAGTCCCGTATC-3′	5′-TTGTAGTTGGGGTGGTCCTG-3′
OCT4	5′-CGAAAGAGAAAGCGAACCAG-3′	5′-AACCACACTCGGACCACATC-3′
GSC	5′-GAGGAGAAAGTGGAGGTCTGG-3′	5′- GCAAGAAAGTAGCATCGTCTG-3′
FGFR2	5′-TCCTGAGGAGCAGATGACCT-3′	5′-CCGAAGGACCAGACATCACT-3′
FGFR1	5′-GCCAGGACCCGAACAGAG-3′	5′-CCCAGAAGAGGAGGCACTT-3′
RARβ	5′-ATGCTGGATTTGGTCCTCTG-3′	5′- TGCACCTTTAGCACTGATGC-3′
FGF4	5′-GACTACCTGCTGGGCATCAA-3′	5′- TGCACTCATCGGTGAAGAAG-3′
PTF1α	5′-GCCATCGGCTACATCAACTT-3′	5′- GGAGGGAGGCCATAATCAG-3′
NGN3*	5′-GCTCATCGCTCTCTATTCTTTTGC-3′	5′- GGTTGAGGCGTCATCCTTTCT-3′
NKX6.1	5′-ATTCGTTGGGGATGACAGAG-3′	5′-CGAGTCCTGCTTCTTCTTGG-3′
ALB	5′-GCAAGGCTGACGATAAGGAG-3′	5′-TGGCTTTACACCAACGAAAA-3′
Raldh2	5′-CACCATGACTTCCAGCAAGA-3′	5′-CAGGGAACACTCTCCCACTC-3′
AFP	5′-CTT TGG GCT GCT CGC TAT GA-3′	5′-TGG CTT GGA AAG TTC GGG TC-3
PROX1	5′-TCACCTTATTCGGGAAGTGC-3′	5′-GTACTGGTGACCCCATCGTT-3′
NKX2.2	5′-TCTACGACAGCAGCGACAAC-3′	5′-GGGTCTCCTTGTCATTGTCC-3′
NKX2.1	5′-ACCAGGACACCATGAGGAAC-3′	5′-CGCCGACAGGTACTTCTGTT-3′
GCG	5′-AGAGGTCGCCATTGTTGAAG-3′	5′-GCAGGTGATGTTGTGAAGATG-3′
INS*	5′-AAGAGGCCATCAAGCAGATCA-3′	5′-CAGGAGGCGCATCCACA-3′

Primer systems marked with an asterix were designed according to D'Amour et al. [7], [33].

#### Immunocytochemistry

hESC were washed once with phosphate-buffered saline (PBS) and then fixed for 15 minutes in 4% paraformaledehyde (PFA) (BDH) at room temperature followed by an additional PBS wash. Cells were permeabilized with 0.5% TritonX-100 (BDH) for 15 minutes, blocked in PBS with 0.1% Tween (Research Organics) (PBS-T) supplemented with 5% normal donkey serum (NDS) (Calbiochem) for one hour at room temperature, and incubated with the primary antibody in blocking buffer over night at 4°C. The following primary antibodies were used: goat anti-Pdx1 (1∶1500) and rabbit anti-PDX1 (1∶1000) (both from C. Wright at Vanderbilt University Medical Center, Nashville, USA), goat anti-FOXA2 (1∶200)(Santa Cruz) (a gift from P. Serup at the Hagedorn Institute, Gentofte, Denmark), rabbit anti-HNF6 (1∶30)(Santa Cruz), rabbit anti-SOX9 (1∶500) (Chemicon International), and rabbit anti-Phospho-Histone H3 (1∶400) (Upstate). The following day, cells were washed once with PBS and then incubated for one hour at room temperature in the dark with the secondary antibody in PBS-T, washed once again, and then incubated with DAPI (1∶1000)(Sigma) for four minutes. The following secondary antibodies were used: Cy3-α-rabbit (1∶1000)(Jackson ImmunoResearch), 488-α-goat (1∶1000) (Molecular Probes/Invitrogen). Images of immunofluorescently stained cells were taken on a Nikon Eclipse TE 2000-U Axioplan 2 fluorescence microscope and AxioVision LE software was used. Images were edited in Adobe®Photoshop version 8.0. For quantification of PDX1-positive cells, cells in five different randomly chosen images from two separate experiments were calculated. Based on these calculations, an average value±SEM was determined.

#### Receptor inhibition

The FGF signaling was inhibited by SU5402 (Calbiochem). SU5402 is a receptor tyrosine kinase inhibitor functioning by competing for the ATP-binding site within the catalytic domain of the receptor. SU5402 targets vascular endothelial growth factor (VEGF) receptors and FGFR1. However, since FGFRs are highly conserved, SU5402 is considered to be a universal inhibitor of FGF signaling [32]. The RA signaling is antagonized by AGN193109, which was synthesized by NovoNordisk and subsequently provided by the P. Serup laboratory at the Hagedorn Institute, Gentofte, Denmark.

### Cell viability assay

AlamarBlue™ (Biosource) was used to assess cell viability according to the protocol supplied.

## Results

### FGF4 and RA direct differentiation of PDX1^+^ cells from Activin A/Wnt3a-treated hESCs

**Figure 2:**
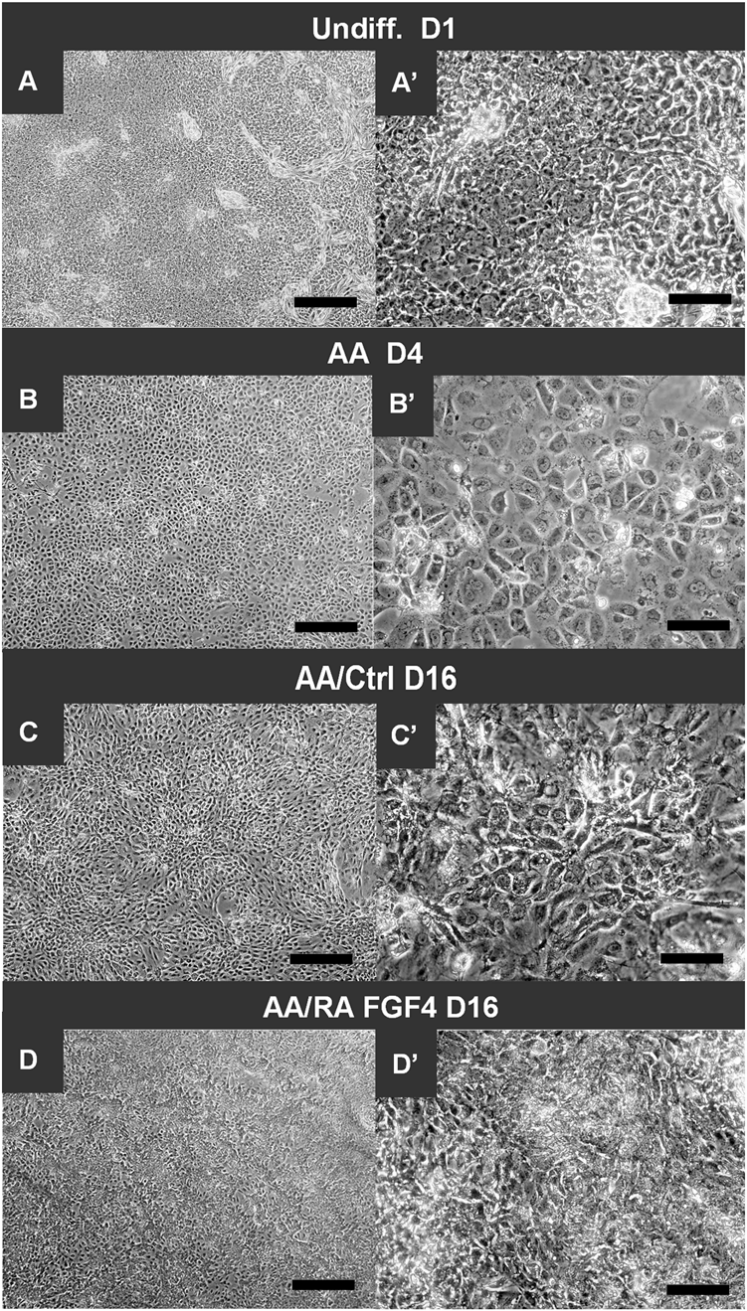
Morphological appearance of cells at different stages of the FGF4/RA differentiation protocol. (A and A′) “Undiff. D1” = Undifferentiated cells at the start of experiment at day 1. (B and B′) Endoderm-like cells after Activin induction (AA) at day 4, (C and C′) Untreated cells (ctrl = control) after Activin induction (AA) at day 16, (D and D′) Cells treated according to the Fgf4/RA differentiation protocol after Activin induction (AA) at day 16. Scale bars: left column, 500 µm; right column, 100 µm.

The pivotal role of RA and FGF4 in endoderm and pancreas development led us to investigate their role in directing differentiation of putative DE, obtained through the frequently used three-day Activin A/Wnt3a induction protocol [33] (AA-induction) (Fig. S1 and S2), into *PDX1* ^+^ posterior foregut endoderm. So far, FGF4 has not been tested for its activity in patterning ESC-derived gut endoderm. In the absence of RA, FGF4 was unable to induce *PDX1* expression (data not shown). Since it was previously shown that RA promotes differentiation into *PDX1* ^+^ cells when added four days after the AA-induction [7], we tested whether FGF4 synergized with RA in directing DE into *PDX1* ^+^ cells. Indeed, *PDX1* expression measured on day twelve increased when FGF4 was added directly after AA-induction and before the RA-treatment (Fig. 1B). Notably, FGF4 exhibited its effect on *PDX1* expression in a concentration-dependent manner. Importantly, endogenous expression of *FGF4* is only detected in undifferentiated cells and not at later time-points (Fig. S3A). To further optimize the protocol, the timing of RA addition was considered. In fact, the timing of RA addition has in most previous efforts been rather arbitrary, based on the fact that it should be added rather soon after the DE-induction. Logically, the timing of RA addition should be based on *RARβ* expression, which so far has not been determined. Therefore, we examined the timing of *RARβ* expression after AA-induction. Interestingly, *RARβ* was upregulated directly after the AA-induction on day four, and subsequently downregulated (day eight) in the absence of any exogenous growth and differentiation factor (GDF) (Fig. 1C and 5A; compare AA D4 with AA D8). Based on these findings we then tested various combinations of FGF4 and RA to achieve optimal induction of *PDX1* expression during a twelve-day period (Fig. 1D). Moreover, *PDX1* expression increased at day 12 when RA was added at day four compared to at day eight (Fig. S3B). Further optimisation of the protocol revealed that the highest *PDX1*-expression level was obtained when RA was kept throughout the whole protocol, i.e. for 13 days after the activin induction. Subtraction of RA at earlier time points (from day 10, 12 or 14) diminished the relative expression of *PDX1* (Fig. S3C). Yet further prolonged treatment with RA and FGF4 still increases *PDX1*-expression, but at this point cells could start to deteriorate, probably due to high confluence. Notably, the marked increase in cell number, but lack of effect on relative *PDX1* expression, upon addition of FGF4 (compare condition 3 with 2 in Fig. 1D) suggests that FGF4 primarily affect cell survival. Moreover, the cell viability assay AlamarBlue indicated that FGF4 promotes cell viability by reducing cytotoxic effects possibly exhibited by RA (Fig. S3D). Based on this observation we show that continuous treatment with RA and FGF4 (1.1 ng/ml) after the AA-induction resulted in efficient induction of *PDX1* mRNA expression (∼25-fold increase in relative *PDX1* mRNA expression on day 13; Fig. 1D). Immunofluorescence analysis was used to confirm that the observed increase in *PDX1* mRNA expression was paralleled by a significant increase at the protein level (Fig. 1E). It should be noted that in cells that did not receive treatment with RA and FGF4, no PDX1-protein was detected. Efforts were made to passage the cells to new plates at this stage, but under currently used experimental conditions the cells failed to survive this treatment. The effect of RA and FGF4 was also evident by changes in cell morphology. Treatment with RA and FGF4 resulted in smaller cells that often were assembled in small cell clusters (Fig. 2).

**Figure 3:**
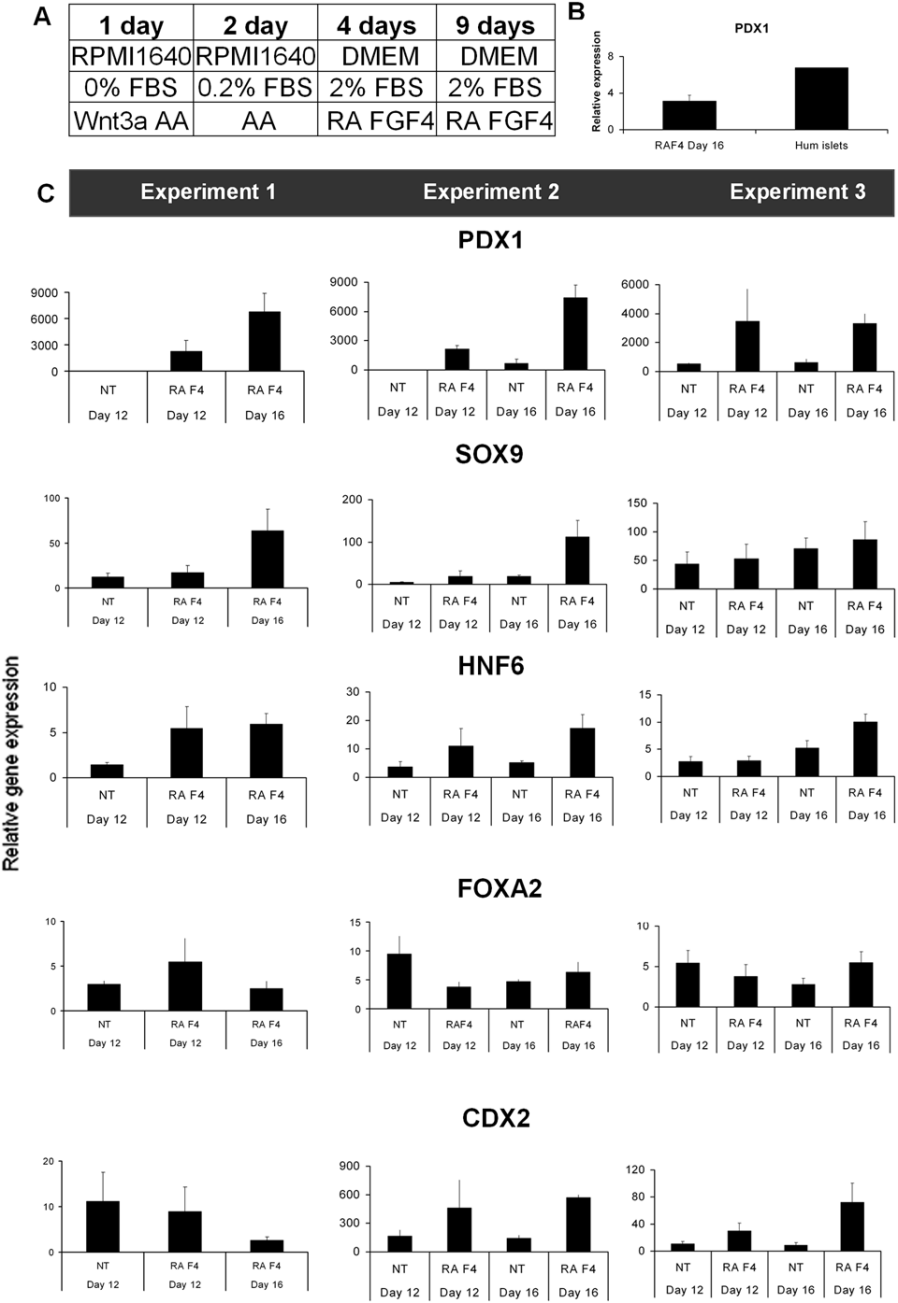
Gene expression analysis of gut endodermal markers at day 16 using the FGF4/RA differentiation protocol. (A) The FGF4/RA differentiation protocol. FBS = fetal bovine serum. Activin = Activin A 100 ng/mL, Wnt3a (25 ng/mL). (B) Relative mRNA expression of PDX1 in RA/FGF4-treated hESC (Day 16) and human islets (C) Relative mRNA expression of *PDX1*, *FOXA2*, *HNF6*, *SOX9*, and *CDX2* at day 12 and 16 with or without (NT = no treatment) addition of RA and Fgf4 (F4) after AA-induction. In these experiments cell line Hues-3 (subclone 52) was used. In Experiment 1, NT day 16 is missing.

To test the reproducibility of the combined action of RA and FGF4 to direct differentiation of *PDX1*-expressing cells, we repeated our protocol (Fig. 3A) three times using cell line Hues-3 (subclone 52) at different passages. More specifically, passage 68, 75 and 76 were used. In order to get some relevant estimation of the magnitude of *PDX1*-expression in differentiated hESC at day 16, the expression was compared to *PDX1*-expression in human islets. *PDX1*-mRNA levels in differentiated hESC were approximately 50% of the levels detected in human islets (Fig. 3B). In order to analyze the real-time PCR data, the lowest value of each data set was set to one and all other values were related to this. Following this procedure, a mean value was calculated for each of the duplicate or triplicate samples. In some cases, the non-treated cells did not have any measurable level of *PDX1*-transcripts (Fig. 3C; Experiments 1 and 2) and consequently Ct-values were set to 45. Moreover, to further establish the robustness of this protocol, its cell line specificity was tested. For this purpose, the RA/FGF4 protocol was tested on another hESC line: Hues-15 (Fig. S2). Indeed, RA/FGF4 effectively induced *PDX1* expression in Hues-15 (Fig. S2B). Thus, the fact that RA and FGF4 significantly increased *PDX1* mRNA expression in Hues-15 and Hues-3 subclone 52, suggests that the ability of these factors to direct differentiation of AA-induced hESC into *PDX1* ^+^ cells is cell line independent.

When the D'Amour protocol [7] was tested on cell line Hues-1, cells died at stage three. However, with cell line Hues-3, a small number of PDX1^+^ cells was obtained at stage three. Still, cells did not survive further treatment onto stage four and five (data not shown). Importantly, these PDX1 expression levels were never as high as with our RA/FGF4-protocol.

### FGF4 and RA direct differentiation of hESCs into *PDX1* ^+^ foregut endoderm

In order to determine whether the induced *PDX1* ^+^ cells represents posterior foregut pancreatic endoderm or non-pancreatic foregut endoderm, the expression of markers characteristic for such cell types were examined. Whereas the general gut endoderm marker *FOXA2* was expressed at high levels at all time points and unaffected by RA/FGF4-treatment, the effect on expression of the midgut/posterior gut endoderm marker *CDX2* varied (Fig. 3B). Consistent with the increase in *PDX1* mRNA expression, a corresponding increase in the transcription of the foregut endoderm markers *HNF6* and *SOX9* was observed (Fig. 3B). However, mRNA expression of markers characteristic of posterior foregut pancreatic endoderm, such as *PTF1α* and *NKX6.1* was very low, suggesting that the combined action of RA and FGF4 results in induction of *PDX1* ^+^ foregut endoderm. In addition, mRNA expression of NKX2.2, NKX2.1, Glucagon (GCG) and Insulin (INS) was also very low or undetectable. Thus, we speculate that the cells obtained with our protocol represent multipotent foregut endoderm with the potential to become pancreatic, posterior stomach, or duodenal endoderm.

Control cells, i.e. cells that subsequent to the AA-induction were not treated with FGF4 and RA, adopted a hepatic fate as determined by an upregulation of liver progenitor/hepatocyte marker expression, including albumin, α-fetoprotein (AFP) and prospero-related homeobox-1 (PROX1) (Fig. S4A).

**Figure 4:**
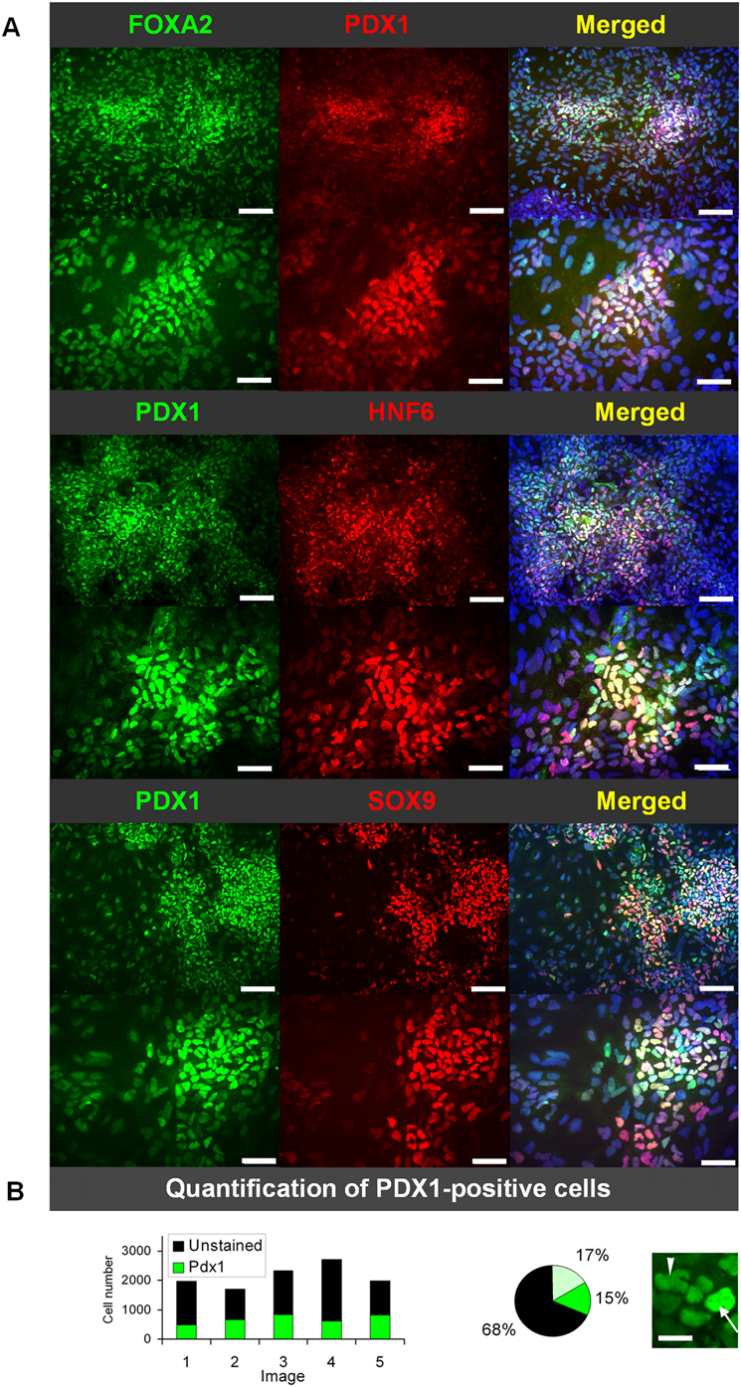
Characterization of PDX1^+^ cells generated by the Fgf4/RA differentiation protocol on day 16. (A) Immunofluorescence stainings of PDX1, SOX9, FOXA2, and HNF6. Nuclei are indicated by DAPI staining. Scale bars: 100 µm. (B) Quantification of the total amount of PDX1-positive cells on day 16 (bar chart) and low and high-intensity PDX1-positive cells (pie chart). A cell representative of high PDX1-expression is indicated by an arrow and of low PDX1-level by an arrow head. Scale bar: 10 µm.

To more directly examine the nature of the *PDX1* ^+^ cells, immunofluorescence stainings with antibodies against gut endoderm, foregut endoderm, and posterior pancreatic foregut endoderm were performed. All PDX1^+^ cells, which primarily were found in clusters, expressed FOXA2 (Fig. 4A). Furthermore, a predominant fraction of the PDX1^+^ cells co-expressed HNF6 and SOX9 (Fig 4A). Altogether, these data are consistent with the Q-PCR data and supports the notion that FGF4 and RA effectively direct differentiation of foregut endoderm.

Sonic hedgehog (Shh) signaling suppresses induction of a pancreatic fate both in vivo and in vitro during ESC differentiation [34]–[36]. However, preliminary results indicate that blocking SHH signaling with cyclopamine at different time-points have no impact on the appearance of *PDX1* ^+^ endoderm (data not shown). We also investigated if RA had a direct downregulating effect on *SHH*, which has been reported in Zebrafish studies [37], [38]. However, this was not the case.

PDX1^+^ cells were quantified from five randomly chosen images from two experiments. In each image, 2000–3000 cells were analysed. Based on this analysis, the fraction of PDX1^+^ was estimated to be 32.5%±3.7%. In addition, the PDX1^+^ cells were classified as low-expressing (17%) or high-expressing (15%) (Fig. 4B). Notably, the levels of PDX1-expression showed no correlation with the expression of any of the other examined endoderm markers.

Immunofluorescence detection of phospho-histone H3 demonstrated that very few Pdx1^+^ cells replicated on day sixteen of the FGF4/RA protocol (Fig. S4B).

### RA signaling is necessary for PDX1 induction

**Figure 5:**
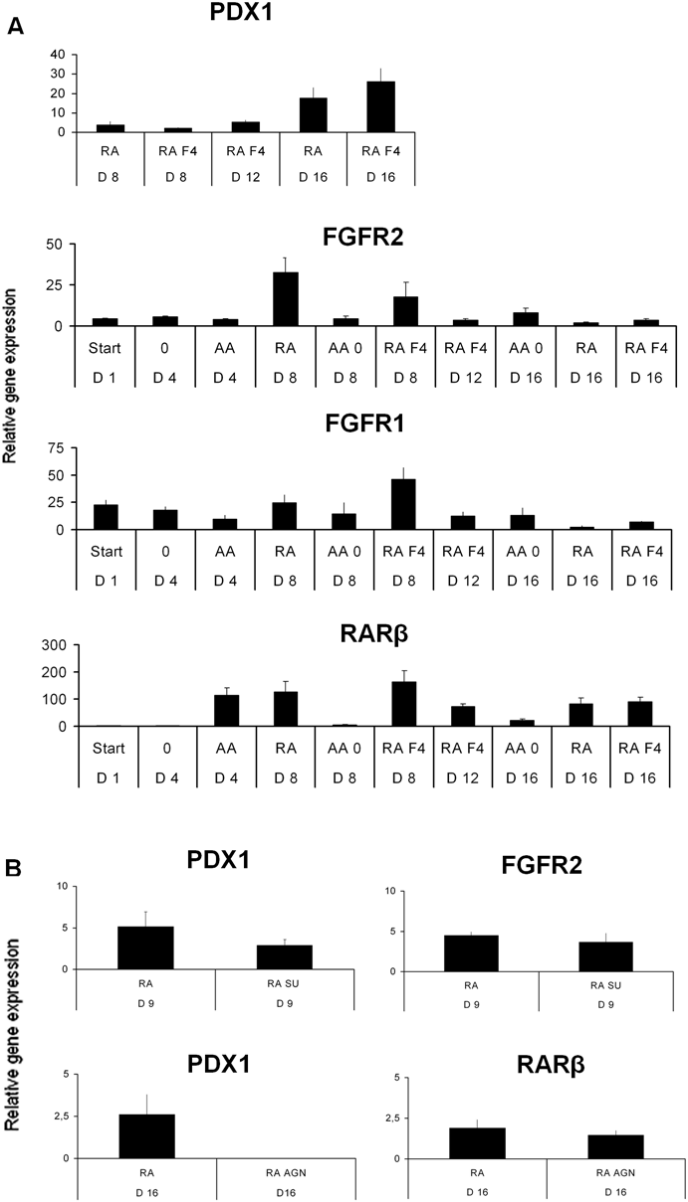
Analysis of expression and role of FGF and RA receptors during the FGF4/RA differentiation protocol. (A) Relative mRNA expression of *PDX1*, fibroblast growth factor receptors 1 and 2 (*FGFR1*, *FGFR2*), and retinoic acid receptor beta (*RARβ*) on different days (D1–D16) during the induction. RA, retinoic acid; F4, fibroblast growth factor 4. Cells were always pretreated with Activin-A (AA)-induction for three days. (B) Pharmacological inhibition of FGF (SU)- and RA (AGN)-receptor signaling on day nine of the FGF4/RA differentiation protocol. SU, SU5402 at 5 ng/mL; AGN, AGN193109 at 1 µM; 0 = no addition of any substance; AA, activin A; AA0, AA the first three days followed by no addition of FGF4/RA. Data is based on three separate experiments with cell line Hues-3 (subclone 52).

RA and FGF4 signaling coordinate anterior-posterior patterning of the gut endoderm [24], [39], [40]. Moreover, both RA and FGF signaling is of key importance in induction of PDX1^+^ cells in the foregut endoderm [15], [41]. Raldh2 is neither expressed by non-treated cells nor by RA/FGF4-treated cells at any stage of the protocol, suggesting that endogenous RA is not prevalent in this system. This observation is consistent with the very low to undetectable levels of *PDX1* mRNA in non-treated control cells. In order to begin to elucidate by which mechanism RA and FGF4 promote differentiation into *PDX1* ^+^ cells from hESCs, the temporal expression pattern of *FGFRs* and *RARβ* was examined. Interestingly, *RARβ* transcription was upregulated during the AA-induction (day four), and maintained high in the presence of RA until day eight, after which it declined (Fig. 5A). RA also affected *FGFR* expression. In particular, *FGFR2* expression was upregulated by RA at day eight, after which it declined (Fig. 5A). To more directly determine whether RA is required for *PDX1* transcription, the RA antagonist, AGN193109, was added during days four to fifteen. Indeed, the RA antagonist completely blocked the RA-induced *PDX1* expression (Fig. 5B). The fact that blockage of FGFR signaling (SU5402) in the presence of RA reduced relative *PDX1* mRNA expression at day nine, suggests that at least part of RA's stimulatory effects is mediated by FGF signaling. Thus, the RA added in our protocol may allow FGF4 action by inducing FGFR, although endogenous RA action in mouse embryos may function by repressing FGF signaling. Blocking FGF signaling for longer period compromised cell survival (data not shown). Altogether, these data suggests that early RA signaling is required for induction of *PDX1* expression in AA-induced hESCs, and that at least part of this activity can be explained by FGFR signaling.

## Discussion

RA and FGF4 signaling control endoderm patterning and pancreas induction/expansion in a temporal and concentration-dependent manner in vivo. However, so far the combinatorial role of these GDFs in differentiation of hESCs towards various gut endoderm derivatives has not been tested. In addition, most differentiation protocols towards pancreatic cell types do not provide data on how the optimal concentration and timing of individual GDFs were selected. Here, we provide data on the individual and combinatorial role of RA and FGF4 in directing differentiation of AA-induced hESCs into *PDX1*-expressing cells. By testing out the optimal concentration and timing of adding FGF4 and RA, we show for the first time that RA and FGF4 in a dose-dependent manner synergistically induce differentiation into PDX1^+^ cells (on average 32%). In contrast to the in vivo situation, FGF4 does not influence anterior-posterior patterning of the gut endoderm, but promotes cell survival. Furthermore, we show that RA is required for converting AA-induced hESCs into PDX1^+^ cells, and that part of the underlying mechanism involves FGFR signaling. Finally, further characterization of the PDX1^+^ cells suggests that they represent foregut endoderm. We speculate that these cells represent multipotent foregut endoderm with the potential to become pancreatic, posterior stomach, or duodenal endoderm. Interestingly, activin-treated hESCs that spontaneously differentiate in the absence of exogenous RA and FGF4 adopt a liver fate, as assessed by the expression of AFP, Albumin and PROX1.

RA plays a prominent and conserved role in pancreas specification [14]–[17], [39]. Preceding pancreas formation, RA also regulates pre-patterning of endoderm [15]. Consistent with these findings, RA promotes differentiation of *PDX1*-expressing cells from mESCs and hESCs [7]–[9], [12]. The lack of data on the optimal timing of adding RA to hESCs differentiating towards endodermal derivatives led us to follow the expression-pattern of *RARβ*. We show that the AA-induction upregulates *RARβ* already at day four. Consistently, we find that adding RA directly after the AA-induction results in the most efficient induction of *PDX1* expression.

Dessimoz et al. show that in chick studies, FGF4 induces posterior endoderm markers in a concentration dependent manner and inhibits expression of anterior endoderm markers, such as *Hex1* and *Nkx2.1*. Furthermore, they also demonstrate that moderate levels of FGF4 maintain *Pdx1* expression, whereas high levels of FGF4 signaling repress *Pdx1* expression [24]. However, whether FGF4 exhibits the same activity on pluripotent stem cell-derived endoderm in vitro remains unknown. Here, we tested the role of FGF4 alone and in combination with RA in inducing *PDX1* expression. FGF4 alone was unable to induce *PDX1* ^+^ cells from AA-induced hESCs independent of the concentration used and time of addition (data not shown). Notably, FGF4 exhibited no posteriorizing effect on gut endoderm as determined by markers characteristic for anterior and posterior gut endoderm (data not shown). However, in combination with RA, FGF4 promoted cell survival. Whether FGF4 exhibit additional effects on cell differentiation remains to be determined. Interestingly, the observation that blockage of FGF signaling in the presence of RA reduced relative *PDX1* mRNA expression is consistent with such an activity.

Co-localization studies show that a fraction of FOXA2^+^ cells co-express PDX1, but that all PDX1^+^ cells co-express FOXA2. FOXA2 (HNF3β) is a member of the signaling nuclear factor-3/forkhead family of transcription factors [42], which is expressed in foregut endoderm and the derivatives thereof as well as in some ectodermal and mesodermal tissues [43]. This observation suggests that all PDX1^+^ cells are of a foregut origin. *Foxa2* is co-expressed with the ONECUT transcription factor *Hnf6* (Hepatocyte nuclear factor 6) in the developing pancreatic epithelium [44]. In the mouse embryo, *Hnf6* is expressed in many tissues, among which the developing pancreatic epithelium is one. Since *Foxa2* expression is stimulated by *Hnf6*, it has been proposed that *Hnf6* is a key component in the pancreatic transcription cascade. Moreover, *Hnf6* regulates pancreatic endocrine cell differentiation and controls expression of the proendocrine gene *Ngn3* [45]. In addition, *Hnf6* is required for induction of *Pdx1* expression in the ventral pancreatic bud but not in the dorsal pancreatic bud [45], [46]. We found HNF6 to be expressed in the majority of PDX1^+^ cells, supporting the notion that the predominant fraction of PDX1^+^ cells represents foregut endodermal cells. The caudal related homeobox gene *CDX2*, which is expressed in midgut, posterior gut endoderm as well as in trophectoderm [47], was inconsistently regulated by RA/FGF4. SOX9 is an HMG-box transcription factor that is expressed in multipotential pancreatic progenitors and later in duct cells [48], stem cells and paneth cells of the intestinal epithelium [49], neuronal cells [50], [51], heart [52], and hair [53]. In addition, SOX9 activates expression of the proendocrine marker *Ngn3* and is required for the maintenance of the pancreatic progenitor pool [48], [54]. Moreover, in the developing pancreas, expression of *Sox9* is restricted to PDX1^+^ progenitors and is not found in committed endocrine precursors [48]. We found SOX9 to be expressed in the majority of PDX1^+^ cells. In conclusion, co-localization data show that the RA/FGF4-induced PDX1^+^ cells co-express FOXA2, HNF6, and SOX9, representing foregut endoderm. However, although these markers are expressed in multipotent pancreatic endoderm, their expression in the non-pancreatic foregut endoderm precludes judgement of a pancreatic endodermal phenotype. In order to evaluate whether any of the PDX1^+^ cells represents pancreatic endoderm, expression of *PTF1α (PTF1/p48)* and *NKX6.1* were examined. *PTF1α* is a member of the basic helix-loop-helix (bHLH) transcription factor family that, in addition to being expressed in non-endodermal cell types such as various neuronal precursor cells [55]–[57], is specifically expressed in the early pancreatic endoderm within the foregut endoderm. PTF1α is required for exocrine cell differentiation, and lineage-tracing studies show that *Ptf1α*-expressing cells give rise to all pancreatic cell lineages [58], [59]. NKX6.1, a member of the NK homeodomain transcription factor family, is expressed during mouse fetal development in the majority of pancreatic epithelial cells from the earliest stage of bud formation at E9.5 until the onset of the secondary transition at E13.5 [60]. Thus, PDX1^+^ cells co-expressing PTF1α and NKX6.1 is diagnostic for pancreatic endoderm. However, quantitative analysis of *PTF1α* and *NKX6.1* mRNA expression revealed no, to very low, levels of these mRNAs. Consistently, expression of NKX6.1 protein was undetectable. Thus, in conclusion, the PDX1^+^ cells induced by the RA/FGF4 protocol represent either posterior stomach/duodenal endoderm, or pre-pancreatic posterior foregut endoderm not yet expressing genes representative of pancreatic foregut endoderm.

Multiple points of interactions exist between RA and FGF signaling during embryonic axis formation in *Xenopus* [27] and mouse [26]. The temporally regulated and distinct expression patterns of *RARβ* and *FGFR1*/*FGFR2* led us to test whether RA signaling regulated *FGFR1*/*FGFR2* expression and vice versa. However, blocking FGF signaling had no impact on *RARβ* expression and blocking RA receptors had no impact on *FGFRs* (data not shown). Interestingly, blocking FGF signaling concomitant with RA treatment resulted in reduced relative *PDX1* mRNA expression, supporting the notion that at least part of RA's inductive effect on *PDX1* expression is mediated by FGF signaling. Thus, RA acts partly independent of, and partly synergistically with, FGF signaling in directing differentiation of hESCs into PDX1^+^ foregut endoderm.

In conclusion, we show that RA and FGF4 jointly direct differentiation of PDX1^+^ foregut endoderm in a robust and efficient manner. RA signaling mediated by the early induction of RARβ through AA/Wnt3a is required for *PDX1* expression. Part of RA's activity is mediated by FGF signaling. The differentiation protocol yields on average 32% PDX1-expressing cells representing foregut endoderm. We speculate that these cells represent multipotent foregut endoderm with the potential to become pancreatic, posterior stomach, or duodenal endoderm.

## Supporting Information

Figure S1 Gene expression analysis of Activin (AA) induction using cell line HUES-3 (Subclone 52).(A) The induction protocol. Wnt, Wnt3a; FGF4, Fibroblast growth factor 4; FBS, fetal bovine serum. (B) Relative mRNA expression of CXCR4, goosecoid (GSC), SOX17 and OCT4 at day one (Start) and four (after AA-induction).
(0.32 MB TIF)

Figure S2 Gene expression analysis of the FGF4/RA differentiation protocol at day 16 using cell line HUES-15. (A) Relative mRNA expression of CXCR4, goosecoid (GSC), SOX17 and OCT4 at day one (Start) and four (after AA-induction). (B) Relative expression of PDX1, FOXA2, HNF6, SOX9, and CDX2 at day 12 C (control, no RA/FGF4), 12, and 16.
(0.13 MB TIF)

Figure S3 Endogenous expression of FGF4, early and late requirements of RA and cellular respiration after various RA/FGF4-treatments. (A) Relative expression levels of endogenous FGF4 during the RA/FGF4-differentiation protocol. A = Activin A, RA = retinoic acid, F4 = Fibroblast growth factor 4. (B) Initial requirement of RA. Relative expression of PDX1 after various combinations of RA and F4 from day 4–11. NT = No Treatment. (C) Late requirement of RA. Relative expression of PDX1 after various combinations of RA and F4 from day 4–15. (D) The AlamarBlue assay determines cellular respiration. Fluorescence after various RA/F4-treatments.
(0.48 MB TIF)

Figure S4 Gene expression profile of some liver markers and few proliferating PDX1 cells using the RA/FGF4-differentiation protocol. (A) Relative expression levels of albumin (ALB), α-fetoprotein (AFP), and prospero-related homeobox 1 (PROX1) in non-treated (NT) and RA/FGF4 (RA F4)-treated cells. RA = Retinoic acid, F4 = Fibroblast growth factor 4. Measurements from experiment three are shown. (B) Proliferating cells in mitotic phase indicated by PH3 (phosphor-histone 3)- staining on day 16 of the RA/FGF4-protocol. Arrowheads show PH3/PDX1 double-positive cells. Scale bar: 100 µm.
(1.31 MB TIF)
