# Supplementary figures and images for: FGF4 and Retinoic Acid Direct Differentiation of hESCs into PDX1-Expressing Foregut Endoderm in a Time- and Concentration-Dependent Manner
Source: PLoS One. 2009 Mar 11;4(3):e4794. doi: 10.1371/journal.pone.0004794 (PMC2651644; doi:10.1371/journal.pone.0004794)

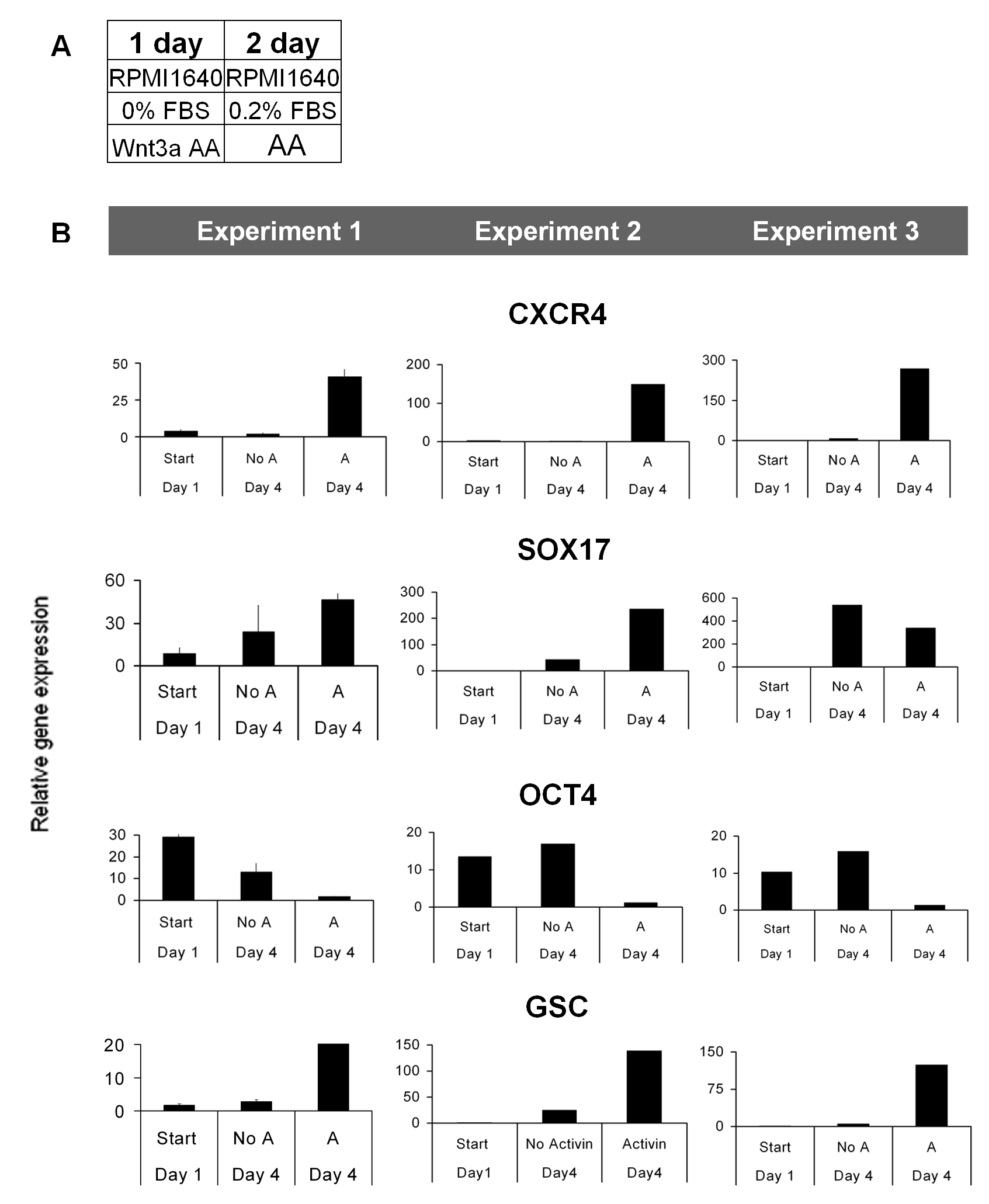

Supplement: Figure S1 — Gene expression analysis of Activin (AA) induction using cell line HUES-3 (Subclone 52).(A) The induction protocol. Wnt, Wnt3a; FGF4, Fibroblast growth factor 4; FBS, fetal bovine serum. (B) Relative mRNA expression of CXCR4, goosecoid (GSC), SOX17 and OCT4 at day one (Start) and four (after AA-induction). (0.32 MB TIF) [file pone.0004794.s001.tif]

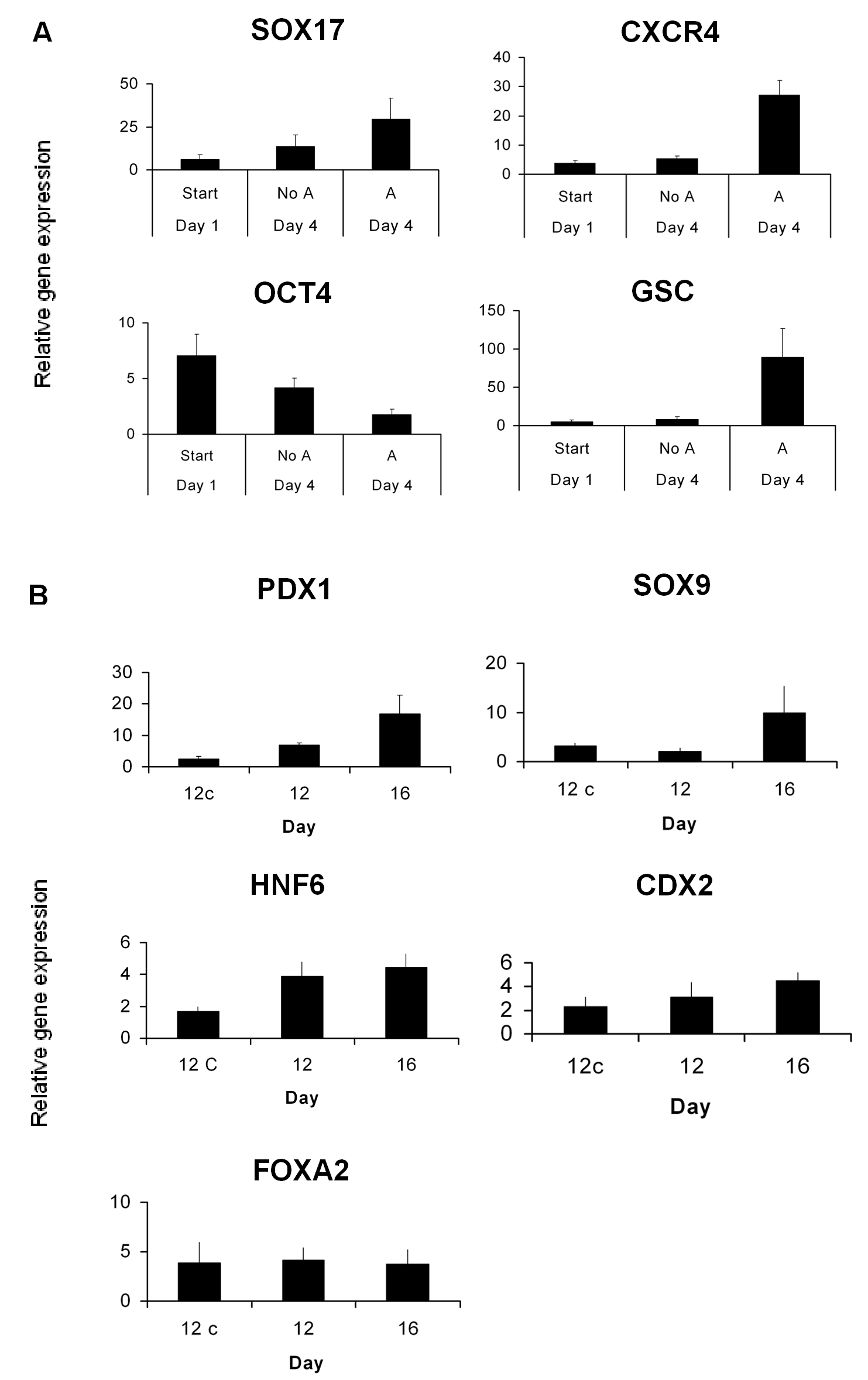

Supplement: Figure S2 — Gene expression analysis of the FGF4/RA differentiation protocol at day 16 using cell line HUES-15. (A) Relative mRNA expression of CXCR4, goosecoid (GSC), SOX17 and OCT4 at day one (Start) and four (after AA-induction). (B) Relative expression of PDX1, FOXA2, HNF6, SOX9, and CDX2 at day 12 C (control, no RA/FGF4), 12, and 16. (0.13 MB TIF) [file pone.0004794.s002.tif]

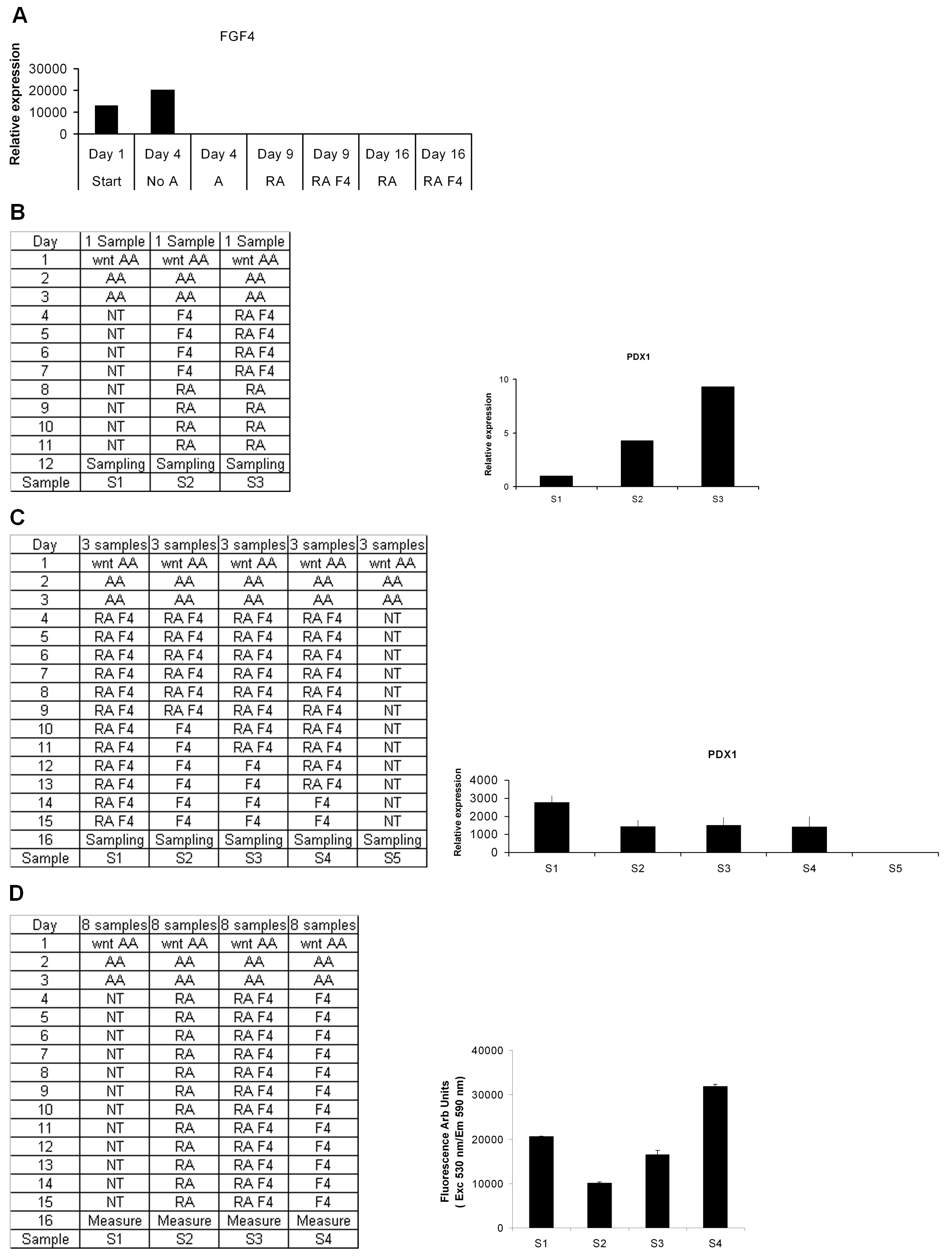

Supplement: Figure S3 — Endogenous expression of FGF4, early and late requirements of RA and cellular respiration after various RA/FGF4-treatments. (A) Relative expression levels of endogenous FGF4 during the RA/FGF4-differentiation protocol. A = Activin A, RA = retinoic acid, F4 = Fibroblast growth factor 4. (B) Initial requirement of RA. Relative expression of PDX1 after various combinations of RA and F4 from day 4–11. NT = No Treatment. (C) Late requirement of RA. Relative expression of PDX1 after various combinations of RA and F4 from day 4–15. (D) The AlamarBlue assay determines cellular respiration. Fluorescence after various RA/F4-treatments. (0.48 MB TIF) [file pone.0004794.s003.tif]

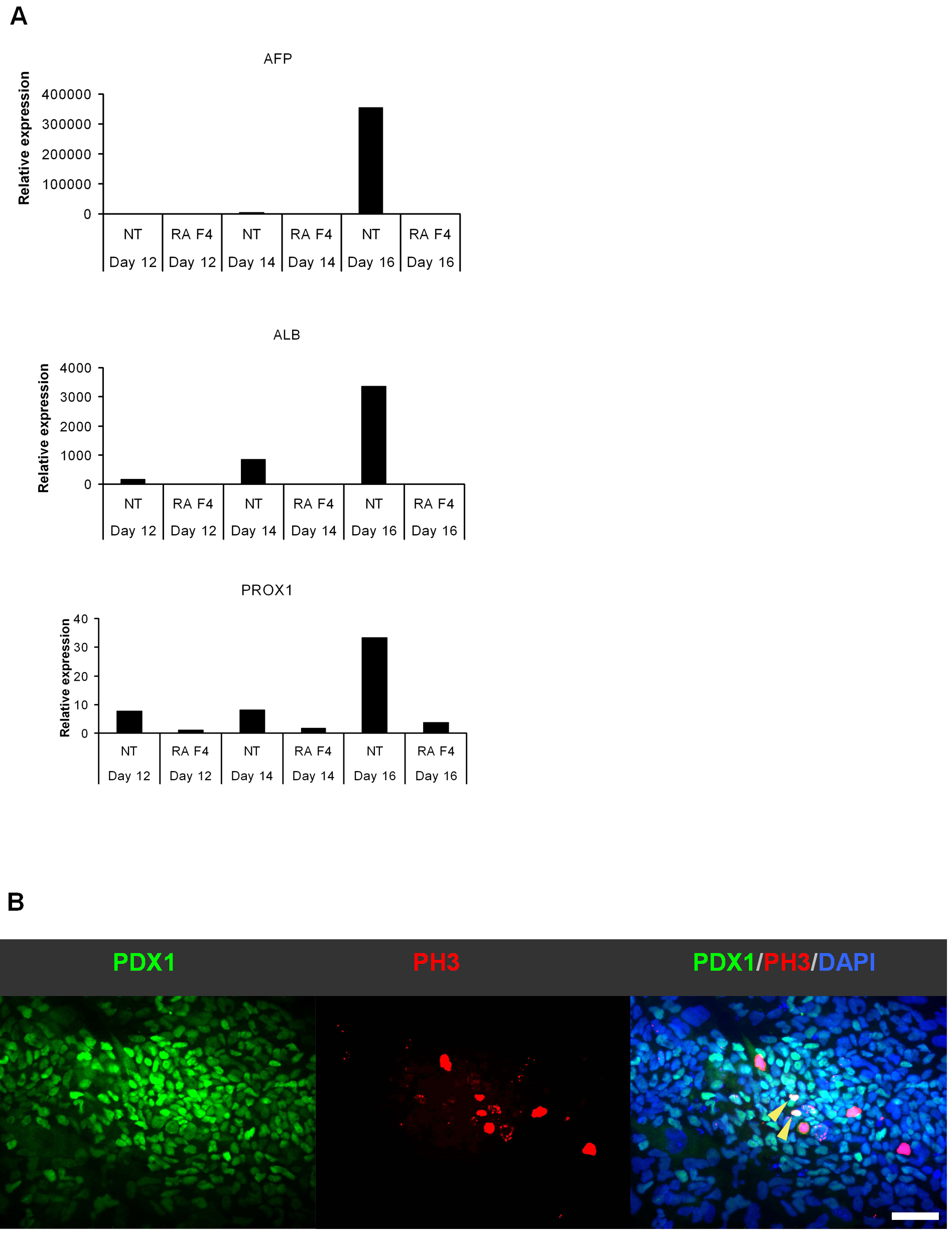

Supplement: Figure S4 — Gene expression profile of some liver markers and few proliferating PDX1 cells using the RA/FGF4-differentiation protocol. (A) Relative expression levels of albumin (ALB), α-fetoprotein (AFP), and prospero-related homeobox 1 (PROX1) in non-treated (NT) and RA/FGF4 (RA F4)-treated cells. RA = Retinoic acid, F4 = Fibroblast growth factor 4. Measurements from experiment three are shown. (B) Proliferating cells in mitotic phase indicated by PH3 (phosphor-histone 3)- staining on day 16 of the RA/FGF4-protocol. Arrowheads show PH3/PDX1 double-positive cells. Scale bar: 100 µm. (1.31 MB TIF) [file pone.0004794.s004.tif]
